# Rectal colonization with multidrug-resistant organisms in adult acute leukemia patients undergoing allogeneic hematopoietic stem cell transplantation: a retrospective, longitudinal, single-center study

**DOI:** 10.3389/fcimb.2026.1902132

**Published:** 2026-10-16

**Authors:** Edoardo Tamellini, Chiara Bonetto, Annarita Mazzariol, Fulvia Mazzaferri, Erica Diani, Francesca Tolio, Sara Pagnin, Alessandra Vatteroni, Angela Crema, Alberto Schena, Cristina Tecchio

**Affiliations:** 1 Department of Engineering for Innovation Medicine, Innovation Biomedicine Section, Hematology and Bone Marrow Transplant Unit, University of Verona, Verona, Italy; 2 Department of Neuroscience, Biomedicine and Movement Sciences, Psychiatry Section, University of Verona, Verona, Italy; 3 Department of Diagnostics and Public Health, Microbiology Section, University of Verona, Verona, Italy; 4 Department of Diagnostics and Public Health, Infective Disease Section, University of Verona, Verona, Italy

**Keywords:** acute lymphoblastic leukemia, acute myeloid leukemia, allogeneic hematopoietic stem cell transplantation, infection-related mortality, multidrug-resistant organisms, overall survival, rectal colonization, transplant-related mortality

## Abstract

**Introduction:**

Rectal colonization with multidrug-resistant organisms (MDROs) represents a frequent issue in patients undergoing allogeneic hematopoietic stem cell transplantation (allo-HSCT) for acute leukemia. However, the prognostic significance of MDRO-colonization in these patients remains debated.

**Methods:**

This retrospective, longitudinal, single-center study included 100 patients with acute myeloid or lymphoblastic leukemia treated at Verona University Hospital from diagnosis to allo-HSCT. MDRO-colonization was monitored by rectal swab at admission and weekly during hospitalization. Colonized patients underwent targeted antibiotic therapy when clinically required.

**Aim of the study:**

To assess epidemiology, timing, and impact of rectal MDRO-colonization on infection occurrence, infection-related mortality (IRM) within day +100, overall survival (OS), and transplant-related mortality (TRM).

**Results:**

Rectal colonization with 44 MDRO strains was detected in 35 (35%) patients undergoing allo-HSCT, either at hospital admission (45.7%) or during hospitalization (54.3%). *Enterobacterales* accounted for 72.7% of MDROs, with 68.5% of colonizing strains being detected during previous hospital stays for induction or consolidation therapies. MDRO-related bloodstream infections (BSI) occurred in 9 (9%) patients, 7 (77.8%) of whom being rectal carriers. IRM within day +100 was null. OS was significantly lower in MDRO-carriers as compared to non-carriers only at univariate analysis. TRM did not differ significantly between the two groups in both univariate and multivariate analyses.

**Conclusion:**

Despite the limitations of a single-center retrospective study, our data show that careful monitoring of rectal colonization and prompt use of targeted antibiotics may temper the negative impact of MDRO-related infections in adult acute leukemia patients undergoing allo-HSCT, even in the setting of a higher rate of BSIs.

## Introduction

Allogeneic hematopoietic stem cell transplantation (allo-HSCT) is a potentially curative treatment for a variety of hematologic malignancies, including high-risk acute myeloid leukemia (AML) and acute lymphoblastic leukemia (ALL) (Khaled et al., 2012; Cieri et al., 2021; Loke et al., 2021) However, the profound immunosuppression induced by conditioning regimens and graft-versus-host disease (GVHD) prophylaxis, compounded by delayed immune recovery, places allo-HSCT recipients at high risk of life-threatening infectious complications (Espinoza et al., 2018; Pereira et al., 2019; Yanir et al., 2022). Colonization with multidrug-resistant organisms (MDROs) has emerged as a frequent issue in allo-HSCT patients. Intestinal colonization with MDROs is increasingly recognized among patients with hematologic malignancies and those undergoing allo-HSCT. Reported risk factors vary according to the colonizing organism, patient population, and local epidemiology. Nevertheless, the most consistently identified factors include previous MDRO carriage, prolonged or repeated hospitalization, prior exposure to broad-spectrum antibiotics, the presence of a central venous catheter, intensive chemotherapy or allo-HSCT, and neutropenia, particularly when profound and prolonged. AML has also been associated with an increased risk of intestinal colonization by carbapenem-resistant *Enterobacterales* (Meschiari et al., 2023; Hu et al., 2024; Luo et al., 2024; Baccelli et al., 2025; Kazakou et al., 2026). Routine screening for MDRO-colonization through periodic rectal swabs is now commonly implemented in hematology and HSCT centers, although its true impact on allo-HSCT outcomes remains uncertain (Cao et al., 2022a; Gonçalves et al., 2024). While colonization is known to increase the risk of early post-transplant MDRO-related infections (Averbuch et al., 2017; Gonçalves et al., 2024), its influence on infection-related mortality (IRM) within day +100, overall survival (OS), and transplant-related mortality (TRM), remains unclear (Mizusawa et al., 2020; Kang et al., 2025; van Leeuwen et al., 2025; Kazakou et al., 2026). Furthermore, the primary risk factors and optimal treatment strategies for MDRO-related infections in this context remain poorly defined (Averbuch et al., 2017; Battipaglia et al., 2019; Corcione et al., 2025). Accordingly, the roles of variables such as underlying hematologic malignancy, antibiotic prophylaxis, diet, colonization timing, MDRO type, conditioning regimen intensity, GVHD occurrence, and neutropenic fever management, require further investigation.

Among patients undergoing allo-HSCT, those affected by acute leukemia experience prolonged pre-transplant hospitalizations for induction and consolidation therapies, thus being exposed to an increased risk of MDRO-colonization (Guarnera et al., 2023). Currently, limited data are available regarding the clinical impact of transient rectal colonization detected during pre-transplant hospital stays on allo-HSCT outcome (Kazakou et al., 2026).

In this retrospective longitudinal single-center study, we aimed to assess epidemiology, timing, and impact of rectal MDRO-colonization on allo-HSCT outcomes, including infection occurrence, infection-related mortality (IRM) within day +100, OS, and TRM, in a series of 100 adult patients undergoing allo-HSCT for acute leukemia. A second aim of the study was to verify the clinical relevance of colonizations transiently detected during pre-transplant hospital stays, and no longer detectable at the time of allo-HSCT.

## Patients, materials, and methods

This retrospective single-center study reviewed 100 adult patients with AML or ALL who underwent allo-HSCT at the Hematology Unit of Verona University Hospital between April 2019 and April 2025.

Data were retrieved from hospital records (Trakcare, InterSystems, Italy) upon patients’ informed consent. The study was conducted in accordance with the Helsinki Declaration.

To analyze a homogeneous series and obtain comprehensive information on rectal colonization before allo-HSCT, the study included only patients with acute leukemia who were consecutively treated at Verona University Hospital from the time of induction onward.

No changes occurred in the policy regarding allo-HSCT management in the 2019–2025 period. Patients undergoing allo-HSCT were hospitalized in a dedicated ward of the Hematology Unit under protective isolation in a single room, according to Jacie requirements (McGrath and Jawdat, 2021). During pre-transplant hospitalizations for induction or consolidation treatments, patients were placed under protective isolation in single or double rooms in the Hematology Unit. A low-microbial diet was mandatory during all neutropenic phases of acute leukemia treatment and up to 6 months after allo-HSCT. A two-lumen tunneled central venous catheter was placed in all patients early before the conditioning regimen.

Colonization, defined as the detection of at least one MDRO-positive rectal swab, was assessed at admission and subsequently monitored weekly during hospitalizations (**Supplementary Figure 1**). MDROs were defined according to the European Center for Disease Prevention and Control (ECDC) as microorganisms that are non-susceptible to at least one agent in three or more antimicrobial categories (Aïch et al., 2025).

Bloodstream infections (BSI), pneumonia, urinary tract infections (UTI), and soft tissue infections were defined according to the ECDC HALT-4 protocol case definitions (Aïch et al., 2025). The intensity of the conditioning regimen was defined according to the transplant conditioning intensity (TCI) index (Spyridonidis et al., 2020). GVHD prophylaxis consisted of: rabbit antithymocyte globulins (rATG) (5 mg/kg/day for 3 days), cyclosporin A, and short-course methotrexate in the case of HLA-matched related donors; rATG (10 mg/kg/day for 3 days), cyclosporin A, and short-course methotrexate in the case of HLA-matched unrelated donors; post-transplant cyclophosphamide (50 mg/kg/day for 2 days), cyclosporin and mycophenolate mofetil in case of haploidentical related donors. Acute GVHD (aGVHD) was graded according to the Glucksberg criteria (Glucksberg et al., 1974).

Antimicrobial prophylaxis began at allo-HSCT hospital admission and included ciprofloxacin, acyclovir, posaconazole, letermovir according to donor-recipient CMV serostatus from day +6, and trimethoprim-sulfamethoxazole from day +35. Per institutional guidance, fluoroquinolone antibiotic prophylaxis was administered until neutrophil engraftment (absolute neutrophil count [ANC]> 500/mm^3^). In case of fever, 3 sets of blood culture from peripheral vein and central venous catheters, urine culture, and chest X-ray were performed before empiric antibiotic therapy. Non-colonized patients with neutropenic fever received vancomycin and ceftazidime, while patients with rectal MDRO-colonization (hereafter referred to as MDRO-carriers) were treated according to their antibiotic resistance profile. Antibiotic treatment was discontinued in afebrile patients with negative blood cultures at the resolution of severe neutropenia. In case of MDRO-related BSI, targeted antibiotic therapy was continued for at least 14 days and withdrawn only after 2 consecutive negative blood cultures. Management of non-neutropenic fever was guided by clinical condition, eventually including targeted antibiotics for MDRO-carriers.

As previously stated MDRO screening was performed by rectal swab. The specimen was streaked on selective media ChromoID ESBL (BioMèrieux, France) plus an ertapenem disk (10 µg) and MacConkey agar plus a meropenem disk (10 µg) to detect extended-spectrum beta-lactamases (ESBL)-producing *Enterobacteria*, carbapenemase-producing *Enterobacteria* (CPE), carbapenem-resistant (CR) *Pseudomonas aeruginosa*, and carbapenem-resistant *Acinetobacter* spp. Carbapenemase production was confirmed by the Carba NP rapid test, and ESBL production by the ESBL NDP rapid test. Carbapenemase enzyme type was investigated using the NG-Test Carba 5 (Biotech), an immuno-chromatographic assay that uses a multiplex Lateral Flow Immune Assay (LFIA) to detect NDM, OXA-48, KPC, VIM, and IMP-like. ChromoID VRE was used to detect Vancomycin Resistant *Enterococci* (VRE). Glycopeptide resistance for *Enterococci* was confirmed by Etest (bioMérieux, France).

Clonality assessment of MDROs was performed using the IR Biotyper FT-IR system (Bruker Daltonics, Germany). Colonies were prepared according to the manufacturer’s instructions and analyzed in quadruplicate using the “default” splicing method (wavenumber 1300-800/cm). Spectral data were processed with OPUS v8.2.28 and interpreted by hierarchical clustering dendrograms generated using Euclidean distance and average linkage. For dendrogram analysis, the clustering cutoff was automatically determined using Simpson’s index, and a set of epidemiologically unrelated isolates was included as an external control to enhance the discriminatory power of clustering analysis. The molecular characterization of the ESBL genes (*bla _CTX-M_, bla _TEM_*, and *bla _SHV_*) was performed through PCR (Dallenne et al., 2010).

### Statistical analysis

Continuous characteristics were described by median and range, while categorical characteristics were given by frequency distribution. The comparison between the MDRO-carriers and non-carriers was performed by a non-parametric Mann-Whitney U test in the case of continuous variables, and Fisher’s exact or Chi-square test, where appropriate, in the case of categorical variables. Survival analysis was performed to evaluate time-to-event outcomes. OS was calculated from the day of allo-HSCT (day 0) to the last follow-up or death, TRM accounted for all deaths, excluding those caused by disease relapse or progression, and IRM within day +100 was defined as death that occurred as a direct consequence of MDRO-related infectious complications. Survival functions were estimated using the Kaplan–Meier method and compared between MDRO-carriers and non-carriers using the log-rank test. Median survival times and corresponding 95% confidence intervals (CIs) were reported where applicable. To explore the association between covariates and the event hazard, univariable and multivariable Cox proportional hazards regression models were fitted. Results were expressed as hazard ratios (HRs) with 95% CIs. Variables included in the multivariable model were selected based on p-values <0.10 in the univariable analysis. The threshold was chosen to avoid excluding potentially important candidate variables while maintaining a parsimonious model (Harrell, 2015; Chowdhury and Turin, 2020). All tests were two-sided and were considered significant if p-values < 0.05. Statistical analyses were performed using Jamovi 2.6.44.

## Results

### Patients’ characteristics

The study enrolled 100 patients affected by AML (74%) and ALL (26%), with a median age of 58 (range 21-75) years. Unrelated HLA-matched donors were the most frequent (69%), followed by related HLA-matched (22%) and haploidentical (9%) donors. Eighty-seven patients received a myeloablative conditioning regimen with an intermediate or high TCI score. No significant difference was detected in baseline characteristics between MDRO-carriers and non-carriers (**Table 1**).

**Table 1:** Patients’ and allo-HSCT characteristics.

	Total	MDRO +	MDRO -	p
Patients	100	35	65	-
M/F	55/45	20/15	35/30	0.402^°^
Median age, years (range)	58 (21-75)	58 (23-75)	58 (21-75)	0.565^§^
Acute leukemia				
**Myeloid**	76	26 (74.3%)	50 (79.9%)	0.809^°^
**Lymphoblastic**	24	9 (25.7%)	15 (23.1%)	
Donor type				
**Matched related**	22	10 (28.6%)	12 (18.5%)	0.506^#^
**Haploidentical**	9	3 (8.6%)	6 (9.2%)	
**Matched unrelated**	69	22 (62.8%)	47 (72.3%)	
HCT-CI score				
**0 (low)**	56	19 (54.3%)	37 (57%)	0.967^#^
**1-2 (intermediate)**	30	11 (31.4%)	19 (29.2%)	
**>3 (high)**	14	5 (14.3%)	9 (13.8%)	
Disease status				
**Complete remission**	91	30 (85.7%)	61 (93.8%)	0.271^°^
**Active disease**	9	5 (14.3%)	4 (6.2%)	
TCI score				
**1-2 (low)**	13	2 (5.7%)	11 (16.9%)	0.257^#^
**2.5-3.5 (intermediate)**	78	29 (82.9%)	49 (75.4%)	
**4-6 (high)**	9	4 (11.4%)	5 (7.7%)	
aGVHD > grade 2	26	17 (26.2%)	9 (25.7%)	1.000^°^

HCT-CI score: Hematopoietic Cell Transplantation-Specific Comorbidity Index score

TCI: Transplant Conditioning Intensity

^°^ Fisher’s exact test; ^§^ Mann-Whitney U; ^#^ Chi-square test

### Pattern and timing of MDRO-colonization in patients undergoing allo-HSCT

Thirty-five of 100 patients undergoing allo-HSCT were colonized with 44 MDRO strains based on rectal swab analysis: 16 (45.7%) patients were MDRO-colonized at hospital admission, while 19 (54.3%) tested positive within day +33 post-allo-HSCT (i.e. maximum 40 days after hospital admission).

Among the 21 MDRO strains isolated at hospital admission, 14 (66.7%) were Gram-negative, all being *Enterobacterales*, mostly ESBL-producing *Escherichia coli* and ESBL-producing *Klebsiella pneumoniae*, while Gram-positive strains were 7 (33.3%), mostly represented by *Enterococcus faecium* VRE (**Table 2**).

**Table 2:** MDRO strains detected in patients undergoing allo-HSCT.

STRAIN	ALLO-HSCT		TOTAL n=44
	ADMISSION n=21	HOSPITAL STAY n=23	
GRAM-NEGATIVE	14 [12]	21 [12]	35 [24]
*Enterobacterales*	14 [12]	18 [10]	32 [22]
• ESBL ***E. coli***	7* [6]	7* [4]	14** [10]
• 3CG-R ***E. cloacae***	1 [1]	2 [0]	3 [1]
• ESBL ***K. pneumoniae***	5** [4]	5 [3]	10** [7]
• CR ***K. pneumoniae***	1 [1]	2 [2]	3 [3]
• ESBL ***K. oxytoca***	–	1 [1]	1 [1]
• ESBL ***C. freundii***	-	1 [0]	1 [0]
*Others*	–	3 [2]	3 [2]
• MDR ***S. maltophilia***	-	2 [1]	2 [1]
• MDR ***B. cenocepacia***	–	1 [1]	1 [1]
GRAM-POSITIVE	7 [6]	2 [1]	9 [7]
• VR **E. *faecium***	6 [5]	2 [1]	8 [6]
• VR **E. *faecalis***	1 [1]	-	1 [1]

[ ] Square brackets enclose the number of strains isolated during previous hospital stays.

ESBL, Extendend Spectrum Beta-Lactamase; 3CG-R, Resistant to Third-Generation Cephalosporins; CR, Carbapenem Resistant; MDR, multidrug-resistant; VR, Vancomycin Resistant

*Single strain acquiring carbapenem resistance

Among the 23 MDRO strains isolated during the hospital stay, within day +33 post-allo-HSCT, 2 were identified as additional colonizing strains in 2 patients who were known as MDRO-carriers since hospital admission, while 21 strains were identified in 19 patients who became MDRO-carriers during the hospital stay (**Table 2**). Twenty-one (91.3%) out of 23 MDRO strains were Gram-negative, with *Enterobacterales* accounting for 18 (85.7%) of isolates. On the contrary, Gram-positive MDROs (both VRE) were only 2 (8.7%) (**Table 2**).

Overall, 35 (79.5%) of 44 colonizing MDROs were Gram-negative, mostly *Enterobacterales* (n=32, 91.4%). Importantly, 2 patients colonized with ESBL-producing *Escherichia coli* and 2 patients colonized with ESBL-producing *Klebsiella pneumoniae* later changed their antimicrobial susceptibility patterns, becoming CR. Three *Klebsiella pneumoniae* strains were CR since the first isolation. Nine (20.5%) of colonizing MDRO strains were Gram-positive, of which 8 (88.9%) were VRE.

The availability of microbiological data of the entire series, from the first hospitalization onward, enabled us to retrieve patients’ previous rectal MDRO-colonization.

Accordingly, among the 21 MDRO strains identified by rectal swab at hospital admission for allo-HSCT, 18 (85.7%) were isolated in the same patients during previous hospitalizations, with the first identification occurring during the hospital stay for induction treatment in 9 (50%) cases, and across the second, third or fourth hospitalization in 6 (33.3%), 2 (11.1%), and 1 (5.6%) case, respectively.

Regardless of the rectal swab negativity at allo-HSCT hospital admission, 13 out of 23 (56.5%) strains detected throughout hospitalization were previously identified at least once in the same patients during the first (8, 61.5%), second (1, 7.7%), third (1, 7.7%), or fourth (3, 23.1%) hospital stay. These MDRO strains recurred on average 13.5 days (2–33) after allo-HSCT, mostly during the neutropenic phase, which had a median length of 17 (11–56) days.

Therefore, only 13 (29.5%) strains were isolated for the first time during hospital stay for allo-HSCT, while the remaining 31 (70.5%) were detected during previous hospitalizations (**Table 2**).

We were able to perform clonality assessment of isolates from pre- and allo-HSCT rectal swabs of two patients. The first one tested positive for ESBL-producing *Escherichia coli* during the second hospital stay and remained continuously positive until the post-transplant phase (**Supplementary Figure 2**). The PCR confirmed the presence of *bla*_CTX-M1_ group ESBLs.

The second one resulted colonized with ESBL-producing *Klebsiella pneumoniae* at a single rectal swab performed during the second hospital stay, testing positive again for the same strain during the neutropenic phase post-HSCT (**Supplementary Figure 3**). The PCR confirmed the presence of *bla*_SHV_ ESBLs.

In both cases, clonality analysis and PCR confirmed that the distinct isolates originated from the same strain (**Supplementary Figures 2**, **3**).

### MDRO-colonization and infection development

Thirty-eight BSIs were documented, with 9 (24%) of them being MDRO-related, and sustained by ESBL-producing *Klebsiella pneumoniae* (n=5), ESBL-producing *Escherichia coli* (n=1), *Enterobacter cloacae* resistant to third-generation cephalosporins (3GCR) (n=1), MDR *Pseudomonas aeruginosa* (n=1), and methicillin-resistant *Staphylococcus epidermidis* (MRSE) (n=1). MDRO-related BSI occurred in 7 (20%) of 35 carriers *vs* 2 (3.1%) of 65 non-carriers (p=0.005). MDRO-related BSI occurred with a median time of 6.5 days (1–69) after allo-HSCT. No patient required intensive care unit (ICU) intervention.

Eleven MDRO-related UTI were documented, 4 being sustained by ESBL-producing *Escherichia coli*, 2 by ESBL-producing *Klebsiella pneumoniae*, 1 by CR *Klebsiella pneumoniae*, 1 by 3GCR *Enterobacter cloacae*, 1 by *Stenotrophomonas maltophilia*, 1 by CR *Pseudomonas putida*, and 1 by VRE. These infections occurred in 7 (20%) carriers *vs* 4 (6%) non-carriers (p=0.03).

Five skin-soft tissue MDRO-related infections were documented, 2 being sustained by 3GCR *Enterobacter cloacae*, 1 by ESBL-producing *Klebsiella pneumoniae*, 1 by MDR *Staphylococcus aureus*, and 1 by MDR *Corynebacterium* spp. These infections occurred in 3 MDRO-carriers (8.6%) *vs* 2 (3.1%) non-carriers (p=ns), all being sustained by the colonizing strains in the first group of patients.

Pneumonia occurred in 10/35 (28.6%) carriers *vs* 15/65 (23.1%) non-carriers (p=ns). No patient required bronchoalveolar lavage, thus lacking an accurate identification of the causative agent.

### Clinical impact of recurrent MDRO strains

There was no significant difference among recurrent (n=13) vs newly acquired (n=10) post-allo-HSCT strains vs strains detected at hospitalization (n=21) in terms of frequency MDRO-induced BSIs (p=ns) and UTIs (p=ns) (**Supplementary Table 1**).

### Survival analysis

The median length of hospital stay was 32 (20–112) days in MDRO-carriers vs 30 (19–68) days in non-carriers (p=ns). IRM within day +100 was null in both MDRO-carriers and non-carriers.

We considered MDRO-colonization status a time-fixed covariate because the 19 patients who became carriers during their hospital stay were colonized by 13 recurrent and 10 newly acquired strains within day +33 post-allo-HSCT (i.e. maximum 40 days post hospital admission). This occurred with a steady weekly distribution, and small category sizes (**Supplementary Table 2**).

By considering OS, the total number of deaths was 32. Survival curves (based on Kaplan-Meier estimates) of MDRO-carriers and non-carriers differed significantly (log-rank test p=0.012) (**Figure 1**). In detail, the estimated probability of survival for non-carriers vs MDRO-carriers was 84.3% vs 62.9% beyond 12 months, 80.6% vs 53.4% beyond 24 months, and 72.1% vs 49.6% beyond 36 months. MDRO-carriers had 2.37 (95% CI 1.18-4.74, p=0.015) times the risk of experiencing the event compared to non-carriers.

**Figure 1:**
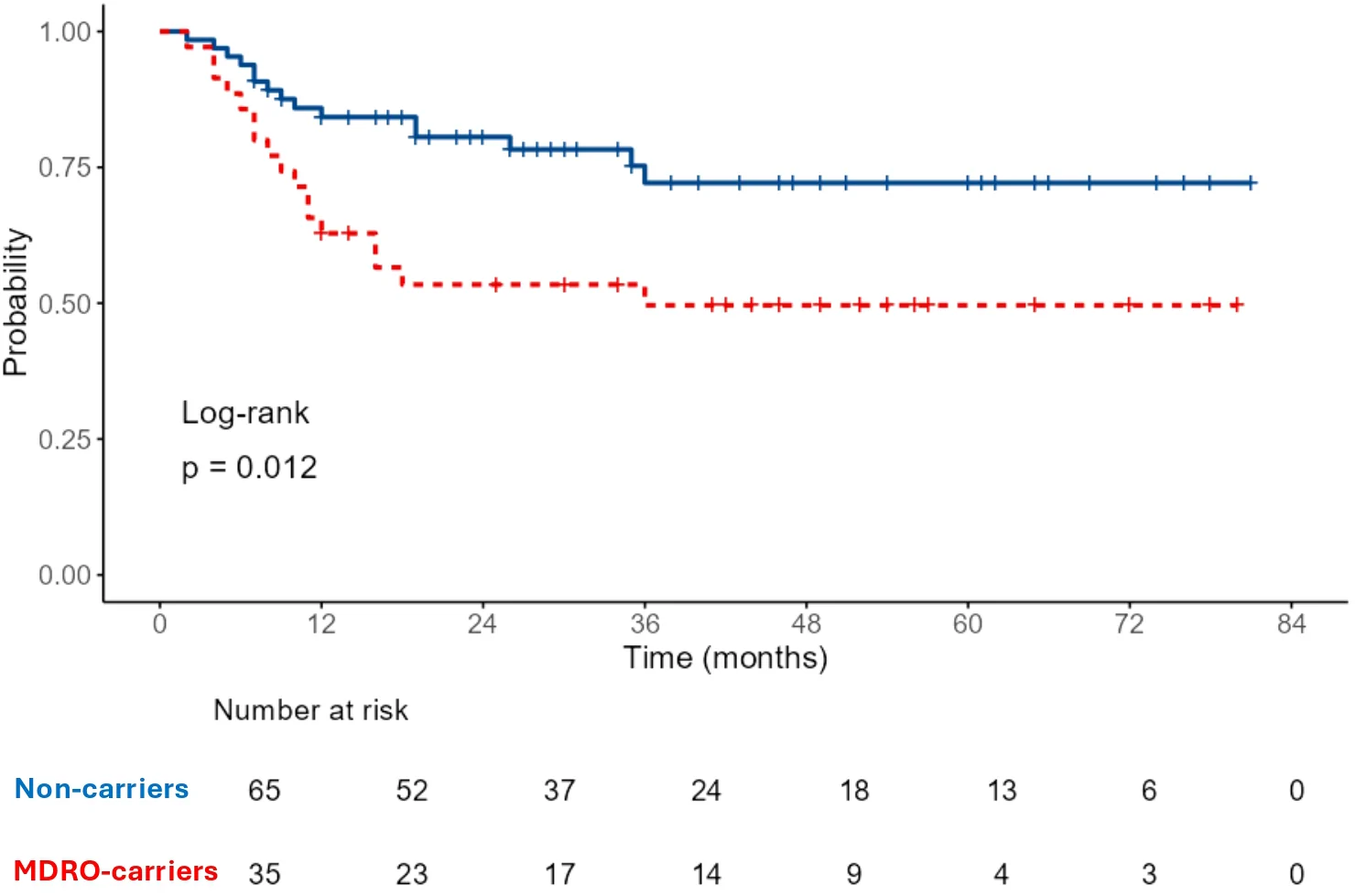
Overall Survival of MDRO-carriers and non-carriers.

By considering TRM, the total number of deaths was 15. Survival curves of MDRO-carriers and non-carriers showed a trend towards significance (log-rank test p=0.075) (**Figure 2**). The comparison of the estimated probability of survival for non-carriers vs MDRO-carriers was 89.0% vs 79.4% beyond 12 months, 89.0% vs 70.6% beyond 24 months, and 84.6% vs 70.6% beyond 36 months. MDRO-carriers had a non-significant HR of 2.45 (95% CI 0.89-6.75, p=0.084) compared to non-carriers.

**Figure 2:**
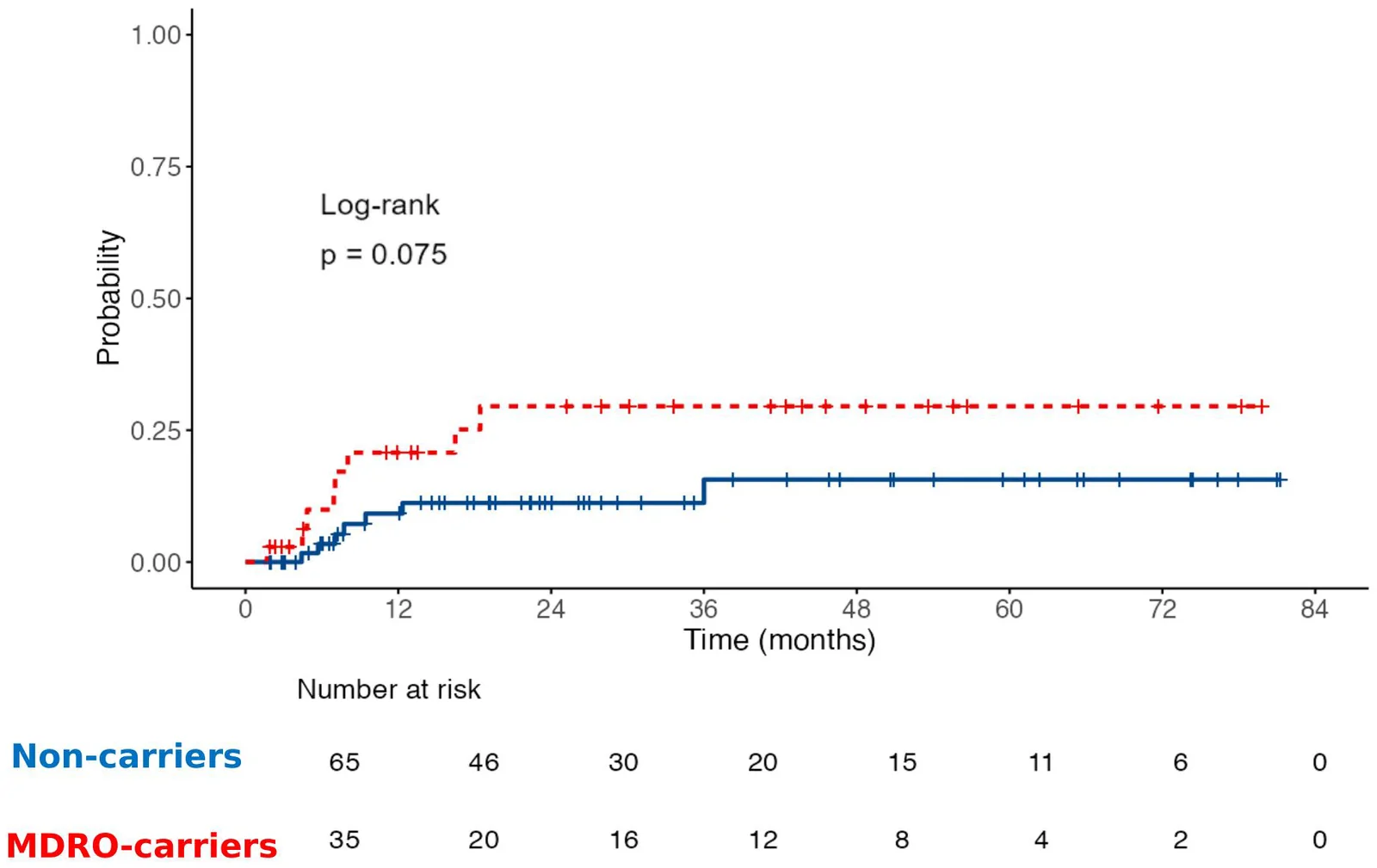
Transplant Related Mortality of MDRO-carriers and non-carriers.

Beside MDRO-carrier status, for both OS and TRM univariable analyses we considered the following characteristics: disease status (complete remission vs active), sex (M vs F), age (<50 vs ≥50 years), donor type (haploidentical vs matched unrelated vs matched related), HCT-CI (low risk vs intermediate risk vs high risk), TCI (low vs intermediate vs high), and acute GVHD (0–1 grade vs 2–4 grade).

As for OS, only MDRO-carrier status and active disease were significantly associated (p=0.015 and p<0.001, respectively) and were included in a multivariable analysis, which showed only disease status as statistically significant (HR 5.11, 95% CI 2.14-12.20, p<0.001), possibly due to the small number of subjects and events (**Table 3**).

**Table 3:** Univariable and multivariable analysis for OS.

	n (%)	HR (univariable)	HR (multivariable)*
MDRO			
Non-carriers	65 (65.0)	–	–
Carriers	35 (35.0)	2.37 (1.18-4.74, p=0.015)	1.73 (0.82-3.65, p=0.148)
Disease status			
Complete remission	91 (91.0)	–	–
Active	9 (9.0)	6.54 (2.90-14.78, p<0.001)	5.11 (2.14-12.20, p<0.001)
Sex			
Male	55 (55.0)	–	
Female	45 (45.0)	1.15 (0.58-2.31, p=0.686)	–
Age			
<50 years	30 (30.0)	–	
>50 years	70 (70.0)	1.67 (0.72-3.87, p=0.229)	–
Donor type			
Haploidentical	9 (9.0)	–	
Matched unrelated	69 (69.0)	0.43 (0.16-1.15, p=0.091)	
Matched related	22 (22.0)	0.55 (0.18-1.69, p=0.295)	–
HCT-CI			
Low risk	56 (56.0)	–	
Intermediate risk	30 (30.0)	1.60 (0.75-3.41, p=0.228)	
High risk	14 (14.0)	1.58 (0.57-4.37, p=0.375)	–
TCI			
Low	13 (13.0)	–	
Intermediate	78 (78.0)	0.90 (0.31-2.61, p=0.848)	
High	9 (9.0)	1.80 (0.48-6.71, p=0.382)	–
Acute GVHD			
0-1 grade	74 (74.0)	–	
2-4 grade	26 (26.0)	1.61 (0.78-3.34, p=0.201)	–

HCT-CI score: Hematopoietic Cell Transplantation-Specific Comorbidity Index score; TCI: Transplant Conditioning Intensity.

*Only independent variables significant at p<0.10 in the univariable analysis were considered for the multivariable one.

As for TRM, only MDRO-carrier status and active disease were significantly associated (p=0.084 and p=0.001, respectively), and were included in a multivariable analysis, which gave only disease status as statistically significant (HR 5.34, 95% CI 1.42-20.05, p=0.013) (**Table 4**). Again, the small sample size and limited number of events may have rendered the model underpowered.

**Table 4:** Univariable and multivariable analysis for TRM.

	n (%)	HR (univariable)	HR (multivariable)*
MDRO			
Non-carriers	65 (65.0)	–	–
Carriers	35 (35.0)	2.45 (0.89-6.75, p=0.084)	1.55 (0.48-4.99, p=0.458)
Disease status			
Complete Remission	91 (91.0)	–	–
Active	9 (9.0)	6.95 (2.20-22.03, p=0.001)	5.34 (1.42-20.05, p=0.013)
Sex			
Male	55 (55.0)	–	
Female	45 (45.0)	2.40 (0.82-7.03, p=0.110)	–
Age			
<50 years	30 (30.0)	–	
>50 years	70 (70.0)	3.08 (0.69-13.65, p=0.139)	–
Donor type			
Haploidentical	9 (9.0)	–	
Matched unrelated	69 (69.0)	0.43 (0.16-1.15, p=0.091)	
Matched related	22 (22.0)	0.55 (0.18-1.69, p=0.295)	–
HCT-CI			
Low risk	56 (56.0)	–	
Intermediate risk	30 (30.0)	0.97 (0.22-4.41, p=0.972)	
High risk	14 (14.0)	1.54 (0.31-7.65, p=0.596)	–
TCI			
Low	13 (13.0)	–	
Intermediate	78 (78.0)	2.29 (0.77-6.82, p=0.136)	
High	9 (9.0)	1.87 (0.26-13.31, p=0.532)	–
Acute GVHD			
0-1 grade	74 (74.0)	–	
2-4 grade	26 (26.0)	1.43 (0.49-4.17, p=0.517)	–

HCT-CI score: Hematopoietic Cell Transplantation-Specific Comorbidity Index score; TCI: Transplant Conditioning Intensity.

*Only independent variables significant at p<0.10 in the univariate analysis were considered for the multivariable one.

## Discussion

The clinical significance of rectal colonization with MDROs in patients undergoing allo-HSCT remains a matter of debate. Over the past decade, several studies have investigated the association between MDRO-colonization and clinical outcome in the allo-HSCT setting, albeit with conflicting results (**Table 5**) (Bilinski et al., 2016; Peric et al., 2017; Scheich et al., 2017; Forcina et al., 2018; Heidenreich et al., 2018; Sadowska-Klasa et al., 2018; Korula et al., 2020; Mizusawa et al., 2020; Cao et al., 2022a; Rogacheva et al., 2022; Santos et al., 2023; Wu et al., 2023; Bono et al., 2025; Kang et al., 2025; van Leeuwen et al., 2025; Kazakou et al., 2026). Most reports documented a higher risk of infection, mostly BSIs, among MDRO-carriers, particularly during the early post-transplant phase. However, as reported in **Table 5**, survival outcomes varied substantially, likely reflecting differences in the pathogenic potential of the colonizing MDRO (Bilinski et al., 2016; Averbuch et al., 2017; Peric et al., 2017; Scheich et al., 2017; Forcina et al., 2018; Heidenreich et al., 2018; Sadowska-Klasa et al., 2018; Chowdhury and Turin, 2020; Mizusawa et al., 2020; Cao et al., 2022a; Rogacheva et al., 2022; Santos et al., 2023; Wu et al., 2023; Bono et al., 2025; Kang et al., 2025; Kazakou et al., 2026). For instance, Sbrana Santos et al., 2023 found no difference in survival when analyzing the overall cohort but observed an increased hazard of death among patients colonized with CR bacteria.

**Table 5:** Literature review: Studies on MDRO-colonization in allo-HSCT patients.

AUTHORS (year)	STUDY DESIGN	STUDY POPULATION number	ENDPOINTS	MDRO-carriers n (%)	RESULTS MDRO+ vs MDRO-	NOTES
Bilinski et al. (2016)	Retrospective, single centre	Allo-HSCT patients (all indications), **107**	BSI rate, NRM, OS	33(31)	BSI: 48% vs 24 (p=0.01) 2y-NRM: 42% vs 11% (p=0.001) 2y-OS: 34% vs 74% (p<0.001)	
Peric et al. (2017)	Retrospective, single centre	Allo-HSCT patients (all indications), **145**	aGVHD incidence, OS	72(50)	aGVHD (III-IV): 27% vs 14% (p=0.04) 2y-OS: 53% vs 49% (p=ns)	
Scheich et al. (2017)	Retrospective, single centre	Allo-HSCT patients with AML, **264**	NRM, OS	142(54)	5y-NRM: 33.9%vs 9.4%(p<0.001) 5y-OS:43.3% vs 65.5%(p=0.002)	
Heidenreich et al. (2018)	Retrospective, single centre	Allo-HSCT patients (all indications), **121**	MDRO persistence NRM, OS	33(27)	2y-NRM: 21% vs 24% (p=ns) 2y-OS: 60% vs 55% (p=ns)	If MDRO persistence >6 months after allo-HSCT: 2y-OS 40% vs 80% (p<0.0001)
Forcina et al. (2018)	Retrospective, single centre	Auto and Allo-HSCT patients (all indications), Allo-HSCT: **254**	IRM, TRM, OS	43(17)	2y-IRM: 23% vs 14% (p=ns) 2y-TRM: 31% vs 25% (p=ns) 2y-OS: 50% vs 43% (p=ns)	
Sadowska-Klasa et al. (2018)	Retrospective, single centre	Allo-HSCT patients (all indications), **120**	IRM, OS	51(43)	IRM: NA 2y-OS:47% vs 64% (p<0.05)	IRM reported higher in MDRO+ patients, data not available
Korula et al. (2020)	Retrospective, single centre	Allo-HSCT patients (all indications), **232**	BSI, 100-mortality	134(58)	BSI: 18.6% vs 7.1% (p=0.01) 100 d-mortality: 29.1% vs 15.3% (p=0.01)	
Mizusawa et al. (2020)	Retrospective, single centre	Allo-HSCT (CBT) patients (all indications) , **145**	BSI, NRM	52(36)	BSI: 30.4% vs 20.7% (p=0.1) 4 y-NRM: 14% vs 2% (p=0.01)	aGVHD as main cause of NRM, higher in MDRO+ patients (31% vs 10%)
Cao et al. (2022)	Retrospective, single centre	Allo-HSCT patients (all indications), **185**	CRE-BSI	44(23)	BSI: 15.9% vs 2.1% (p=0.001)	CRE colonization only
Rogacheva Yu et al. (2022)	Retrospective, single centre	Allo-HSCT patients (all indications), **288**	BSI rate, OS	64(28)	BSI: NA 100 days-OS: 60.6% vs 88.2% (p=0.02)	BSI reported higher in MDRO+ patients (p=0.001), data not available
Sbrana Santos et al. (2023)	Retrospective, single centre	Auto and Allo-HSCT patients (all indications), Allo-HSCT: **343**	BSI, length of hospital stays, OS	132(32.6)	BSI:36.4% vs 20.5% (P=0.002) 100 days-OS: no difference	Colonization with CR-MDRB increase HR for death (4.07, p=0.0005)
Wu QW et al. (2023)	Retrospective, single centre	Allo-HSCT patients (all indications), **343** Screened only for CRE colonization	BSI rate, OS	135(39)	BSI: 11.4% vs 2.6% (p<0.001) 100 days-OS: 90.3% vs 97.6% (p=0.003)	Infection as first cause of death at day 200 in CR+ patients (18/25 vs 8/18)
Van Leeuwen et al. (2025)	Retrospective, single centre	Auto and Allo-HSCT patients (all indications), Allo-HSCT: **124**	BSI	19(15.3)	BSI: 74% vs 49% (P=0.9)	CRE colonization only
Bono et al. (2025)	Retrospective, single centre	Allo-HSCT patients (all indications), **106**	NRM, OS, aGVHD De-escalation approach	34(32)	aGVHD (II-IV): 17% vs 21 (p=ns) 1y-NRM: 15% vs 25% (p=ns) 1y-OS: 72% vs 69% (p=ns)	
Sung-Woon et al. (2025)	Retrospective, single centre	Auto and Allo-HSCT patients (all indications), Allo-HSCT: **182**	BSI	70(33.3)	BSI: 24% vs 2% (p=0.001)	CRE colonization only
Kazakou et al. (2026)	Retrospective, single centre	CAR-T cells, Auto and Allo-HSCT patients (all indications), Allo-HSCT: **155**	BSI rate	61(33)	BSI: 18.7% vs 1.3% (p=NA)	
Present study	Retrospective, longitudinal, single centre	Allo-HSCT patients with acute leukemia, **100**	Day +100 IRM, OS and TRM	35(35)	100-days IRM: null 2y-OS: 53.4% vs 80.6% (p=0.012) 2y-TRM: 29.4% vs 11.0% (p=0.075)	No IRM in the first 100 days No influence of MDRO colonization on OS (univariate and multivariate analysis) and TRM (multivariate)

Allo-HSCT: Allogeneic Hematopoietic Stem Cell Transplantation; BSI: Blood Stream Infection; NRM: Non-Relapse Mortality; OS: Overall Survival; IRM: Infection-related

Mortality; TRM: Transplant-related Mortality; NA: not available; CBT: Cord Blood Transplantation; CRE: Carbapenem Resistant Enterobacteriacae.

In our cohort, colonization by MDROs was identified by rectal swab in 35% of patients, with nearly half being already positive at hospital admission, and the remaining becoming MDRO-positive during hospitalization, after allo-HSCT. ESBL-producing *Enterobacterales* accounted for most of the MDROs detected both at hospital admission and after allo-HSCT. In interpreting this finding, we acknowledge that the routine use of fluoroquinolone prophylaxis until neutrophil engraftment may have influenced the epidemiology of MDRO-colonization in our cohort. Although fluoroquinolone prophylaxis may reduce the incidence of bacterial infections during neutropenia, it may also exert selective pressure on the intestinal microbiota, favoring the persistence or expansion of fluoroquinolone-resistant *Enterobacterales*, including strains harboring additional resistance determinants such as ESBL (Dell’Acqua et al., 2026).

Interestingly, a substantial proportion of the MDROs detected after allo-HSCT had already been documented in the same patients during prior hospital stays, thus suggesting a persistent colonization across treatment phases. In fact, despite a negative rectal screening result at admission, more than half of the MDROs identified after allo-HSCT corresponded to organisms with the same species and resistance phenotype as those previously detected in the same patients. This finding suggests that a single negative rectal swab may not reliably indicate clearance of intestinal colonization. Negative results may reflect intermittent carriage, a bacterial burden below the detection threshold of culture-based screening, sampling variability, or temporary suppression of the colonizing population by antimicrobial exposure. From a clinical perspective, these observations suggest that the longitudinal microbiological history of the patient may be more informative than the most recent screening result. Previous MDRO-colonization should therefore remain part of the individual risk assessment when selecting empirical antibiotic therapy for febrile neutropenia, particularly during the pre-engraftment period and in patients presenting with clinical instability or severe mucosal barrier injury. Nevertheless, a history of colonization should not, by itself, justify the prolonged use of MDRO-directed agents. Empirical treatment should be promptly reassessed and, when clinically appropriate, de-escalated according to the patient’s clinical course, blood culture results, antimicrobial susceptibility data, and local epidemiology. These findings also have organizational implications: microbiological information, including the species identified, resistance phenotype, site and date of isolation, should be systematically transferred between referring hematology units and transplant centers and incorporated into the pre-transplant assessment. Such continuity of information may improve the selection of initial empirical treatment while also supporting timely antimicrobial de-escalation and avoiding unnecessary exposure to broad-spectrum antibiotics.

Consistent with previous literature, this study shows an association between rectal MDRO-colonization and the increased risk of MDRO-related BSI and UTI (Bilinski et al., 2016; Patriarca et al., 2017; Rogacheva et al., 2022; Santos et al., 2023). Specifically, 20% of carriers developed an MDRO-related BSI compared with only 3.1% of non-carriers (p=0.005), and 20% of carriers versus 6% of non-carriers developed an MDRO-related UTI (p=0.03). Notably, none of these infections was life-threatening. Furthermore, there were no CR-bacterial related BSIs; instead, two patients experienced mild CR-related UTIs. Reasonably, the use of targeted empirical antibiotics may have limited the severity of MDRO-related infections. Similar findings were reported by Bono et al. (2025), who adopted a de-escalation strategy, thus addressing the need for a stewardship strategy in the allo-HSCT setting.

As previously stated, no association was found between MDRO-colonization and IRM within day +100. MDRO-carriers showed a significantly lower OS during follow-up at univariate analysis, with most deaths being due to disease progression or TRM unrelated to infectious complications. Accordingly, multivariate analysis showed that MDRO-carriers did not differ from non-carriers with respect to OS and TRM. Therefore, MDRO-colonization may primarily reflect cumulative healthcare exposure, repeated antimicrobial pressure, and greater underlying disease and treatment complexity, rather than acting as an independent determinant of mortality. Nevertheless, the absence of infection-related deaths despite a higher incidence of MDRO-related infections may suggest that systematic surveillance, awareness of previous colonization, and the timely use of individualized empirical antimicrobial therapy contributed to effective infection management. However, this interpretation should remain cautious, as the observational design, the limited sample size, and the low number of severe infectious events preclude any firm conclusion regarding a protective effect of surveillance or tailored antimicrobial strategies. Rather, our results support the potential value of integrating longitudinal microbiological history, current colonization status, clinical severity, and local resistance patterns into empirical treatment decisions, followed by prompt reassessment and de-escalation whenever microbiological and clinical findings allow.

Regarding aGVHD (grade >2), no difference was observed between MDRO-carriers and non-carriers, consistent with Bono et al. (2025), but in contrast with the results of Peric et al. (2017).

We acknowledge that the primary limitations of our study are its retrospective design and relatively small sample size. For instance, due to the study’s retrospective design, we could assess the clonality of recurrent MDROs in only two cases, which nevertheless confirmed the persistence of the same strains over time (**Supplementary Figure 2** and **Supplementary Figure 3**). Moreover, given the limited number of outcome events, the multivariable analyses of OS and TRM should be considered exploratory. The small event-to-variable ratio may have resulted in model overfitting, unstable effect estimates, and limited statistical power to detect independent associations; therefore, the corresponding hazard ratios and confidence intervals should be interpreted with caution. Finally, the single-center design may limit the generalizability of our findings. Outcomes may have been influenced by a center-specific bundle of supportive and infection-control practices, including weekly microbiological surveillance, standardized antimicrobial prophylaxis, colonization-informed empirical antibiotic therapy, dietary precautions, and protective isolation. However, because these interventions were implemented as part of routine care and were not evaluated independently, their individual contribution to the observed outcomes cannot be determined. Multicenter studies with larger cohorts and a greater number of events are needed to validate these observations.

Conversely, the study’s strength lies in the homogeneity of the cohort, which consisted exclusively of patients with acute leukemia treated at our institution since diagnosis, as well as the longitudinal assessment of MDRO-colonization status from disease presentation to allo-HSCT.

In conclusion, our results emphasize the importance of broadening MDRO surveillance beyond admission screening and integrating patients’ past colonization histories into individualized risk assessment and empirical treatment plans. The absence of life-threatening infections within day +100 suggests that a thorough evaluation of concurrent and prior colonization, coupled with prompt initiation of targeted antibiotics, may mitigate the negative impact of MDRO-related infections in adult patients with acute leukemia undergoing allo-HSCT.

## Institutional review board statement

The study was conducted in accordance with the Declaration of Helsinki. Ethical approval was waived because this was a retrospective study analyzing a clinical practice described in SOP (JACIE-accredited center).

## Informed consent statement

Informed consent was obtained from all subjects involved in the study. Consent was waived for deceased patients.

## Data Availability

The raw data supporting the conclusions of this article will be made available by the authors, without undue reservation.

## Supplementary material

Supplementary Table 1 MDRO strains, acquisition time, and MDRO-related infections.

Supplementary Table 2 Patients’ MDRO status, timing of MDRO colonization and distribution of OS and TRM events.

Supplementary Figure 1 Monitoring of MDRO-colonization by rectal swab across treatment phases.

Supplementary Figure 2 Dendrogram obtained by clustering FT-IR spectra for *E. coli*.

Supplementary Figure 3 Dendrogram obtained by clustering FT-IR spectra for *K. pneumonia*.
